# Synergistic Improvement of Strength Characteristics in Recycled Aggregates Using Nano-Clay and Polypropylene Fiber

**DOI:** 10.3390/polym16030374

**Published:** 2024-01-30

**Authors:** Tieyong Zhao, Chenjun Wang, De Zhang, Yanfei Yu, Jiale Luo, Cuihong Li

**Affiliations:** 1 School of Civil Engineering, Shaoxing University, Shaoxing 312000, China; tczhaoty@123.com (T.Z.); wangchenjun63@163.com (C.W.); 2002085045@usx.edu.cn (J.L.); lich@usx.edu.cn (C.L.); 2 Tongchuang and Engineering Design Co., Ltd., Shaoxing 312000, China; 3 Shaoxing Key Laboratory of Interaction between Soft Soil Foundation and Building Structure, Shaoxing 312000, China; 4 Shanghai Road and Bridge Group Co., Ltd., Shanghai 200000, China; dez2021@163.com

**Keywords:** recycled aggregate, nano-clay, polypropylene fiber, unconfined compression strength, deviatoric stress

## Abstract

In order to study the improvement effect of nano-clay and polypropylene fiber on the mechanical properties of recycled aggregates, unconfined compression tests and triaxial shear tests were conducted. The experimental results show that adding polypropylene fibers to recycled aggregates increases the unconfined compressive strength by 27% and significantly improves ductility. We added 6% nano-clay to fiber-reinforced recycled aggregates, which increased the unconfined compressive strength of the recycled aggregates by 49% and the residual stress by 146%. However, the ductility decreased. Under low confining pressures, with the addition of nano-clay, the peak deviatoric stress strength of the fiber-reinforced recycled aggregates first decreased and then increased. When the nano-clay content was 8%, this reached a maximum value. However, under high confining pressures, the recycled aggregate particles were tightly interlocked, so that the improvement effect of the fiber and nano-clay was not obvious. As more nano-clay was added, the friction angle of the fiber-reinforced recycled aggregates decreased, while the cohesion increased. When the content of nano-clay was 8%, the cohesive force increased by 110%. The results of this research indicate that adding both polypropylene fibers and nano-clay to recycled aggregates has a better improvement effect on their strength characteristics than adding only polypropylene fibers. This study can provide a reference for improving the mechanical properties of recycled aggregates and the use of roadbeds.

## 1. Introduction

As large numbers of roads, bridges, houses, and other buildings are constructed all over the world, natural aggregate resources are gradually decreasing [1]. At the same time, the demolition of roads, bridges, houses, and other buildings produces a large amount of construction waste. Landfill or random stacking of construction waste aggregates not only generates a large amount of carbon dioxide, but also causes serious pollution to soil and water resources due to the various ions and Ca(OH)_2_ contained in construction waste [2,3]. The necessity of recycling construction waste has become a focus of global attention [4,5]. After crushing construction waste into suitable-sized recycled aggregates, the recycled aggregates alone cannot fully meet the requirements for road use, so it is necessary to use curing agents or add other geotechnical materials to improve the mechanical properties of recycled aggregates [6] to achieve the purpose of improving their stability and bearing capacity.

Fibers are common soil reinforcement materials, and different types and lengths of fibers have different effects on the mechanical properties of soil. The addition of fibers can significantly enhance the mechanical properties of soil [7,8,9,10,11,12,13]. Hanumesh et al. [14] conducted compressive, shear, and cleavage tests and investigated that up to 50% of recycled aggregates could be used in construction projects. After adding 1–2% polypropylene fibers, the tensile strength, shear strength, and compressive strength of recycled aggregate concrete were significantly improved. Vahid et al. [15] found that the addition of double-headed hook steel fibers could limit the expansion of cracks in recycled aggregate concrete samples and improve their compressive strength. Mohseni et al. [16] found that adding steel fibers and polypropylene fibers together to recycled aggregate concrete could significantly improve their tensile strength, but slightly less than when adding steel fibers alone. Singh et al. [17] found that due to the bridging effect, polypropylene fibers enhance the flexural strength of recycled aggregate concrete, and that the flexural strength of recycled aggregate concrete is directly proportional to the addition of polypropylene fibers. However, if the polypropylene fibers exceed 0.75%, there is no significant impact on the bending resistance [18]. Ahmed et al. [19] found that adding 0.6% polypropylene fibers to recycled aggregate concrete increased the maximum splitting strength by 23.23%. In summary, fibers can not only improve the compressive strength, shear strength, tensile strength, and flexural strength of recycled aggregate concrete, but can also change the crack development and failure mode of soil. However, when the fiber content exceeds a certain threshold, it becomes more difficult for the fibers to be evenly distributed in recycled aggregate concrete, sometimes even forming clusters or spherical shapes. This undoubtedly leads to the formation of more and larger original defects in recycled aggregate concrete, resulting in poor compressive resistance of the recycled aggregate concrete.

Nano-clay particles are widely manufactured using high-energy ball milling machinery and are composed of layered mineral silicates, mainly used as nano fillers and highly active volcanic ash. Nano-clay is an environmentally friendly additive characterized by its extremely high specific surface area and charged active surface, which holds great potential for enhancing soil properties [20,21]. Nano-clay can reduce the workability, porosity, permeability, and water absorption of concrete. This improvement is due to the use of nano-clay particles as nanofillers, nucleation sites, and reactive volcanic ash to promote hydration and enhance concrete performance [22]. Staub et al. [23] conducted a comprehensive study on improving the performance of concrete with nanomaterials. It has been proven that adding different types of nanomaterials can help improve the hardening performance of concrete. Simultaneously, compression, tension, bending, and dynamic strength significantly increase. Iranpour et al. [24] found that the improvement effect of nano-clay on collapsible soil depended on its specific surface area, but excessive addition of nano-clay could lead to particle agglomeration, thereby diminishing the mechanical properties of the soil. Mohsen et al. [25] investigated that under consolidation and drainage conditions, an optimal ratio of glass fiber and nano-clay could improve the peak deviatoric stress and cohesion of sand specimens without altering their internal friction angle. Toghroli et al. [26] found that replacing cement with 1% and 2% nano-clay and 10% silica fume can improve the compressive strength of RAC. Wang et al. [27] studied the compressive strength of self-compacting concrete with a nano-clay content ranging from 0.1% to 0.5%. Their results indicate that the strength of nano-clay concrete increases when the temperature is below 300 °C, and gradually decreases when the temperature is above 300 °C. Ibrahem et al. [28] found that a 10% addition of nano-clay can increase the splitting tensile strength of concrete by 46.8%. In summary, nano-clay can improve the workability, porosity, compressive strength, splitting tensile strength, and other properties of materials such as concrete. However, excessive dispersion defects of nano-clay can lead to a decrease in contact points with the matrix, weakening the interfacial transition zone and having a negative impact on the concrete.

The aforementioned research has demonstrated the modificating effect of fibers and nano-clay on soil and recycled aggregate concrete, but limited attention has been given to investigating the modification effect of these two materials on recycled aggregates simultaneously. This article studies the improvement effect of polypropylene fiber and nano-clay on the mechanical properties of recycled aggregates, including compressive strength, failure mode, and shear strength parameters. The objective is to apply these modified recycled aggregates to roadbed construction.

## 2. Experiment Materials and Scheme

### 2.1. Experiment Materials

**Figure 1:**
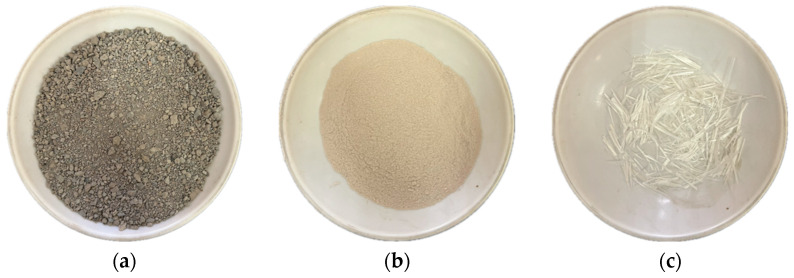
Experiment materials: (**a**) recycled aggregate; (**b**) nano-clay; (**c**) polypropylene fibers.

The recycled aggregate (Figure 1a) used in this experiment is solid waste collected from a road demolition in Shaoxing. Its basic physical properties were tested according to the GB/T 50123-2019 standard [29], as shown in Table 1. The water used in the experiment was tap water. The nano-clay (Figure 1b) used in the experiment was produced by Hubei Jinxi montmorillonite Technology Co., Ltd. (Zhongxiang, China), and the main quality indicators are shown in Table 2. The polypropylene fibers (Figure 1c) used in the experiment were produced by Shaoxing Fiber High Tech Co., Ltd. (Shaoxing, China), with a fiber length of 9 mm, and the parameters are shown in Table 3.

### 2.2. Experiment Scheme

Five mix ratios were designed in the test, as shown in Table 4. Their polypropylene fiber contents were 0% or 0.6% [30,31,32,33] and their nano-clay contents were 0%, 4%, 6%, and 8%. Unconfined compression tests and unconsolidated undrained shear tests were carried out on the five types of specimens. The unconfined compressive strength test specimen is a cylindrical body with a diameter of 50 mm and a height of 50 mm. A fully automatic multi-functional unconfined anti-corrosive press produced by TECO Technology Co., Ltd. (Livermore, CA, USA) was utilized for the test, with the loading rate set at 1 mm/min. The triaxial shear test specimen is a cylinder with a diameter of 39.1 mm and a height of 80 mm, subjected to confining pressures of 100, 200, 300, and 400 kPa, respectively. A fully automatic stress path tester produced by TECO Technology Co., Ltd. was also used, with the loading rate set at 1 mm/min.

### 2.3. Specimen Preparation

**Figure 2:**
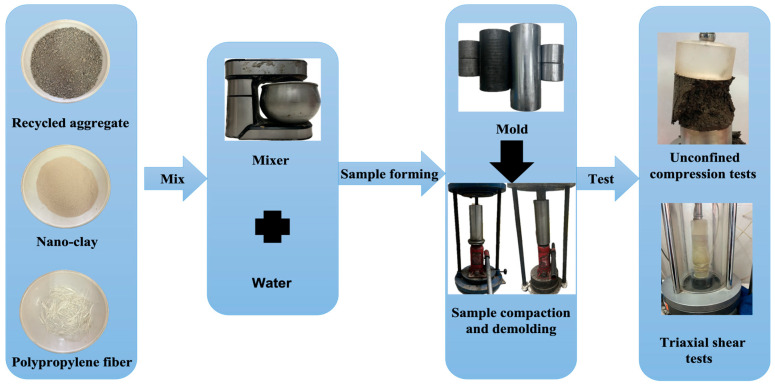
Specimen production and testing process.

As per the Chinese standard JTG E51-2009 [34], the unconfined compressive strength test specimen is specified to be a cylinder with a diameter of 50 mm and a height of 50 mm. Additionally, following the Chinese standard GB/T 50123-2019 [29], the triaxial shear test specimen is designated as a cylinder with a diameter of 39.1 mm and a height of 80 mm. The specimen production and testing process is shown in Figure 2. After the specimen preparation was completed, the unconfined compressive strength test and triaxial shear test were directly carried out. The specimen preparation methods are as follows:

(1) Recycled aggregate treatment. The recycled aggregate block is placed into an oven with a temperature of 105 °C for drying. At the end of the drying process, the fine-grained recycled aggregate used in the test is obtained by removing the large particles from the recycled aggregate block through a 2 mm sieve.
(2) Nano-clay treatment. The nano-clay is dried in a vacuum freeze dryer for 5 h to obtain the nano-clay used in the test.
(3) Mixed mixture. Firstly, the recycled aggregates are mixed with polypropylene fibers and nano-clay until they are evenly blended. Then, water is added, and the mixture is stirred for 6 min. Throughout the mixing process, observations are made every 2 min to ensure that the recycled aggregates, polypropylene fibers, and nano-clay are dispersed as much as possible and clumping is avoided. Finally, after stirring, the mixture is allowed to stand for 2 h [29,34].
(4) Specimen forming. Firstly, the mixture is divided into three equal parts and added to the hollow cylindrical mold. (The unconfined compressive strength test mold has a 65 mm outer diameter, 50 mm inner diameter, and 130 mm height, while the three-axis shear test mold features an outer diameter of 65 mm, an inner diameter of 39.1 mm, and a height of 180 mm.) After each pouring, the mixture should be evenly inserted and vibrated with a tamping rod. Subsequently, a jack is employed to compact the specimen, and the pressure is maintained for 10 min. Finally, utilizing a reaction frame and a jack, the specimen is removed from the mold and shaped into the cylindrical specimens.
(5) Specimen testing. After the sample preparation is completed, there is no need for curing. The unconfined compressive strength test and triaxial shear test are conducted directly [29,34].

To avoid errors caused by the weight and size of the specimen, the mass of the specimen is measured using an electronic balance, and the diameter and height of the specimen are measured using an electronic vernier caliper. Among these, the mass of the specimen cannot exceed ± 5 g of the standard specimen, and the diameter and height errors cannot exceed ± 1 mm.

## 3. Analysis of Unconfined Compression Test Results

### 3.1. Unconfined Compressive Strength

**Figure 3:**
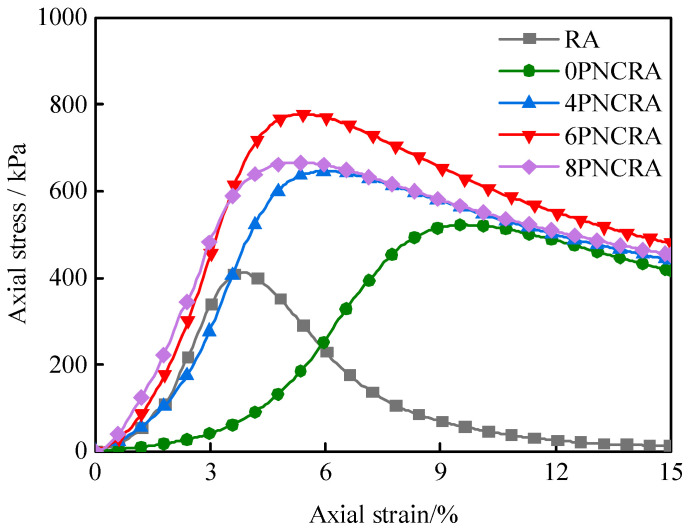
Unconfined compressive strength stress–strain curves of RA and PNCRA.

Figure 3 shows the stress–strain curves of the RA and PNCRA. The calculation formula for axial strain is as follows:

(1) $$\varepsilon =\frac{∆L}{L_{0}}$$

In Equation (1), *ε* represents axial strain, Δ*L* represents the length change of the specimen after being subjected to axial force, and *L*_0_ represents the length of the specimen when not subjected to axial force.

**Figure 4:**
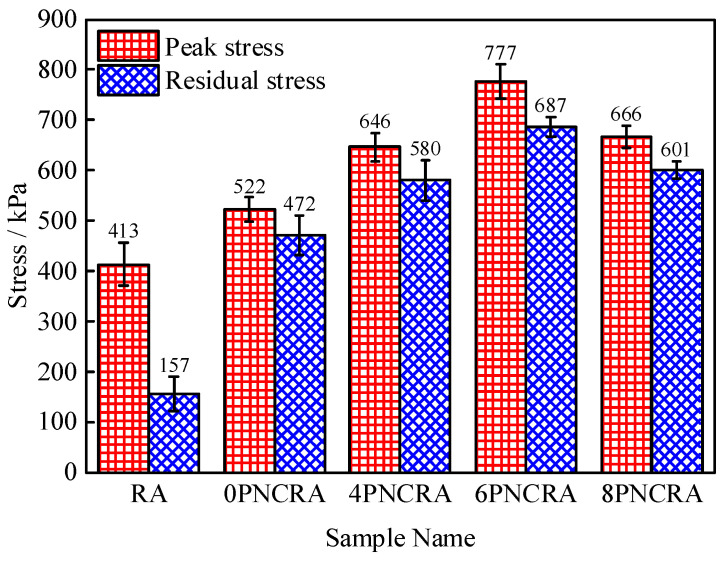
Bar chart of peak stress and residual stress under different nano-clay contents.

The stress–strain curves of the RA and PNCRA under the axial load are softening curves, that is, with increases in the strain, the stress first increases, reaches a peak, then gradually decreases and converges to the residual strength. Figure 4 shows a bar chart of the peak stress and residual stress under different contents of nano-clay. From Figure 4, it can be seen that the unconfined compressive peak strength of the recycled aggregate is increased by 26.6% with the addition of fibers, and the failure mode changes from brittle failure to plastic failure. The unconfined compressive strengths of RA, 0PNCRA, 4PNCRA, 6PNCRA, and 8PNCRA are 413 kPa, 522 kPa, 646 kPa, 777 kPa, and 666 kPa, respectively. With the increase in nano-clay content, the unconfined compressive strength first increases and then decreases under the same fiber content. Compared with 0PNCRA, the unconfined compressive strengths of 4PNCRA, 6PNCRA, and 8PNCRA are increased by 24%, 49%, and 27%, respectively. The 6% content of nano-clay maximizes the unconfined compressive strength. Therefore, it is determined that 6% is the optimal content of nano-clay to enhance the performance the fiber-reinforced recycled aggregate. When the nano-clay content exceeds this optimal level, the unconfined compressive strength decreases. The reason for this is that nano-clay adheres to the surface of fibers and recycled aggregate particles and absorbs water to expand and fill the pores of the fiber-reinforced recycled aggregate, thus improving its compressive strength. However, when the nano-clay content is too high, this process of filling pores will further expand, resulting in the expansion of the pores and the destruction of the original soil structure. An excessive nano-clay content will also cause agglomeration [35], which cannot exert its advantages of large specific surface area, and may even weaken its effect, resulting in a decrease in the compressive peak strength of 8PNCRA [36,37].

### 3.2. Residual Stress

According to the GB/T 50123-2019 [29] specification, the residual stress is selected as 3% of the peak strain. As shown in Figure 4, the residual stresses of 0PNCRA, 4PNCRA, 6PNCRA, and 8PNCRA are 472 kPa, 580 kPa, 687 kPa, and 601 kPa, respectively. As the nano-clay content increases, the residual strain stress of PNCRA first increases and then decreases. Compared to 0PNCRA, the residual stresses of 4PNCRA, 6PNCRA, and 8PNCRA are increased by 123%, 146%, and 127%, respectively. The peak strain gradually decreases with the increase in nano-clay content, and the ductility of the fiber-reinforced recycled aggregate can be described by the ductility index *D* [38].

(2) $$D=\frac{∆fiber}{∆nofiber}$$

In Equation (2), ∆*fiber* refers to the strain corresponding to the peak stress of the recycled aggregate with added fibers, and ∆*nofiber* refers to the strain corresponding to the peak stress of the recycled aggregate without added fibers.

The ductility indexes of 0PNCRA, 4PNCRA, 6PNCRA, and 8PNCRA are 2.49, 1.53, 1.36, and 1.36, respectively. Compared with the other three groups of specimens, the ductility index of the 0PNCRA specimen is significantly higher, indicating that the incorporation of fibers significantly improved the ductility of the recycled aggregate. However, the incorporation of nano-clay reduces the ductility of the fiber-reinforced recycled aggregate, but it is still improved compared with RA. Fibers are randomly distributed in the recycled aggregate, forming a three-dimensional network structure that limits the movement of particles and improves the toughness of the recycled aggregate [39,40]. After the incorporation of nano-clay, the ductility of the specimen decreases, which is due to the fact that after mixing with fibers, nano-clay adheres to the fibers, which prevents the mutual pulling between the fibers without restraint [41]. The ductility of 6PNCRA and 8PNCRA further decreases and remains unchanged because when the nano-clay content is 6%, the fibers are completely covered by nano-clay, and the increase in the nano-clay content no longer affects the ductility.

### 3.3. Failure Mode

**Figure 5:**
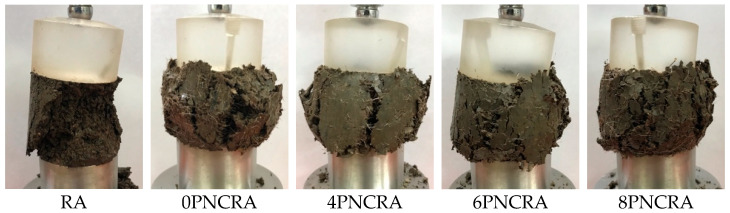
Failure forms of RA and PNCRA.

The damaged specimens are photographed and analyzed from the perspective of damage forms. As can be seen from Figure 5, RA showed obvious shear surface failure during the failure process, and part of it was crushed. There were cracks around 0PNCRA, and these cracks continued to expand. The failure mode of 4PNCRA was similar to that of 0PNCRA, and the recycled aggregate remained in the bond state after failure. Compared with 0PNCRA and 4PNCRA, 6PNCRA and 8PNCRA gradually showed a main surface failure with fewer cracks, and could maintain the failure mode without crushing after failure.

The failure modes of these specimens indicate that fibers can reinforce recycled aggregate, limit its deformation, and improve its toughness and failure mode [42,43]. With the addition of nano-clay, the failure mode gradually shifts from surrounding cracks to a single main surface failure, improving the interface effect of the recycled aggregate [35]. From the above analysis, it can be seen that the addition of fibers and nano-clay can improve the failure mode of recycled aggregates.

## 4. Analysis of Triaxial Shear Test Results

### 4.1. Deviatoric Stress

**Figure 6:**
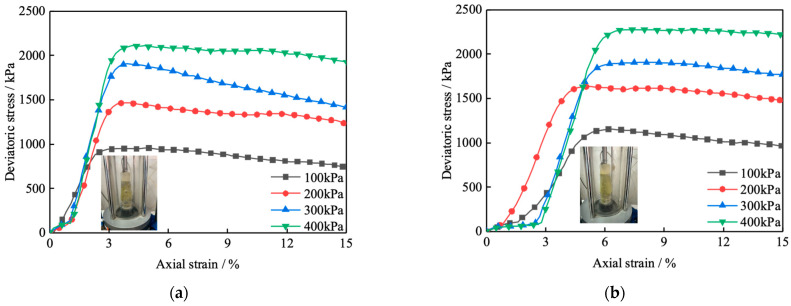

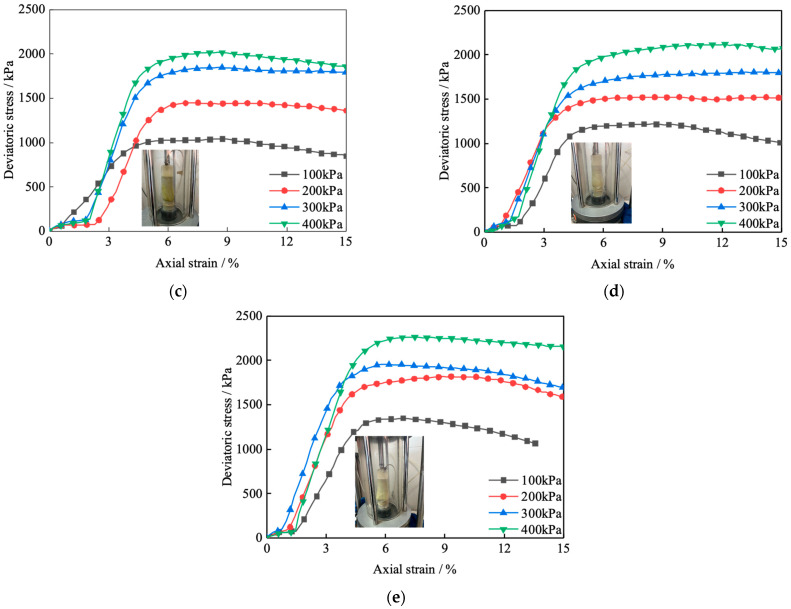
Deviatoric stress–strain curves of RA and PNCRA: (**a**) RA; (**b**) 0PNCRA; (**c**) 4PNCRA; (**d**) 6PNCRA; (**e**) 8PNCRA.

**Figure 7:**
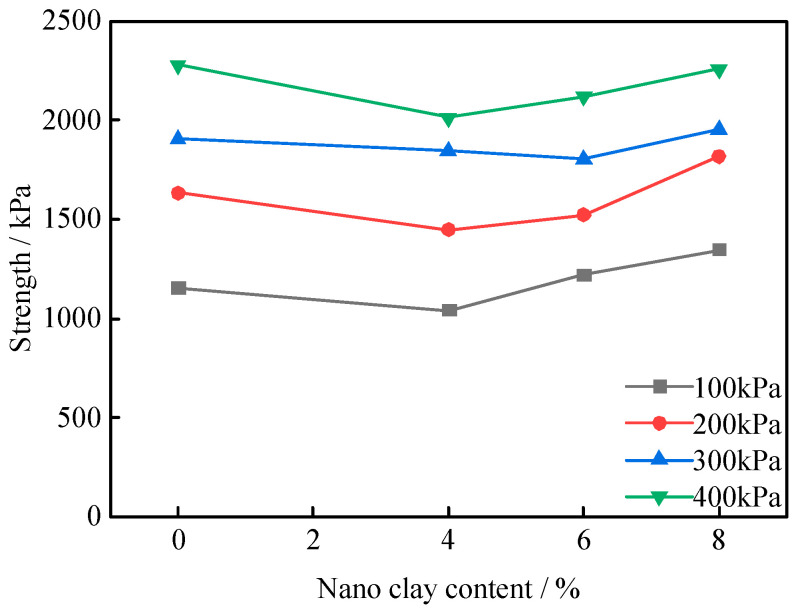
Curve of peak shear strength with the change in nano-clay content.

Figure 6 shows the deviatoric stress–strain curves of RA and PNCRA at 100 kPa, 200 kPa, 300 kPa, and 400 kPa, respectively. It can be seen from Figure 6 that the deviatoric stress–strain curves of RA and PNCRA both exhibit a softening trend. According to GB/T 50123-2019 standard [29], the maximum deviatoric stress is selected as the peak stress point, and the curve of deviatoric stress with the variation in nano-clay content is plotted in Figure 7. At a confining pressure of 100 kPa, the peak deviatoric stress strengths of 0PNCRA, 4PNCRA, 6PNCRA, and 8PNCRA are 1152 kPa, 1040 kPa, 1218 kPa, and 1346 kPa, respectively, which are 20%, 9%, 27%, and 41% higher than that of RA, which is 957 kPa. At a confining pressure of 200 kPa, the peak deviatoric stress strengths of 0PNCRA, 4PNCRA, 6PNCRA, and 8PNCRA are 1633 kPa, 1448 kPa, 1523 kPa, and 1818 kPa, respectively, which are 15%, 2%, 7%, and 28% higher than that of RA, which is 1422 kPa. At a confining pressure of 300 kPa, the peak deviatoric stress strengths of 0PNCRA, 4PNCRA, 6PNCRA, and 8PNCRA are 1907 kPa, 1843 kPa, 1804 kPa, and 1954 kPa, respectively, which are 0%, 3%, and 5% lower than that of RA, which is 1908 kPa. At a confining pressure of 400 kPa, the peak deviatoric stress strengths of 0PNCRA, 4PNCRA, 6PNCRA, and 8PNCRA are 2277 kPa, 2012 kPa, 2118 kPa, and 2257 kPa, respectively, which are slightly lower than that of RA, which is 2111 kPa.

Under the confining pressures of 100 kPa and 200 kPa, the peak deviatoric stress of the fiber-reinforced recycled aggregate decreases first and then increases with the increase in nano-clay content, but it is still higher than that of RA. Under the confining pressures of 300 kPa and 400 kPa, the deviatoric stress still decreases first and then increases with the increase in nano-clay content. Except that 8PNCRA is stronger than RA under both confining pressures, the other groups fluctuate slightly with no significant improvement. Under high confining pressures, the recycled aggregate particles are tightly engaged, and the friction characteristics between particles dominate, so the nano-clay and fibers have a small impact on the strength of the specimen. The confining pressure limits the lateral displacement of recycled aggregate and nano-clay particles, and fibers cannot play a pulling role, resulting in little change in the strength [44].

### 4.2. Shear Strength Parameters

**Figure 8:**
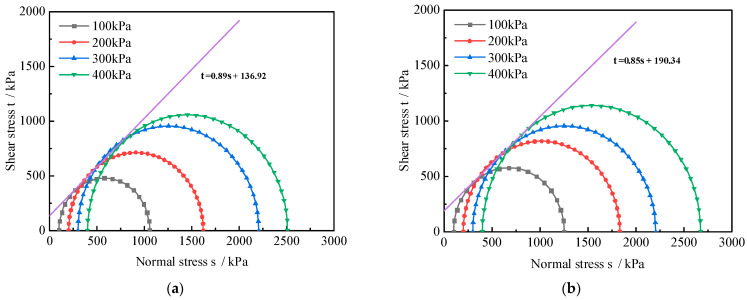

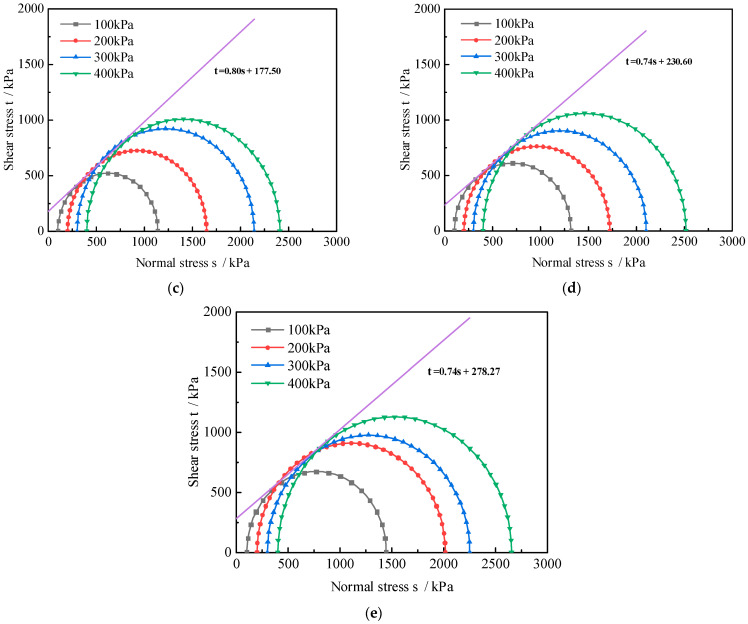
Strength envelope of RA and PNCRA: (**a**) RA; (**b**) 0PNCRA; (**c**) 4PNCRA; (**d**) 6PNCRA; (**e**) 8PNCRA.

As shown in Figure 8, the strength envelopes of RA and PNCRA are drawn according to the Mohr–Coulomb theory, and the deviatoric stress peak intensity Δσ is taken as the failure point, where Δσ = σ_1_ − σ_3_, with the normal stress σ as the abscissa and the shear stress τ as the ordinate. On the horizontal axis, (σ_1_ + σ_3_)/2 is taken as the center and (σ_1_ − σ_3_)/2 is taken as the radius, and the Mohr stress circle is plotted on the stress plane diagram. The shear strength parameters c and φ are calculated through the Mohr stress circle, as seen in Table 5.

According to Table 5, the internal friction angle of 0PNCRA is 40.4°, which is 1.5° lower than that of RA. As the nano-clay content increases, the internal friction angle gradually decreases. Compared to RA, the internal friction angles of 4PNCRA, 6PNCRA, and 8PNCRA are decreased by 3.0°, 5.1°, and 5.3°, respectively. Compared with RA, the cohesive forces of 0PNCRA, 4PNCRA, 6PNCRA, and 8PNCRA are increased by 43%, 34%, 74%, and 110%, i.e., 190 kPa, 178 kPa, 231 kPa, and 278 kPa, respectively.

In summary, the incorporation of fibers into recycled aggregates can improve their cohesion, but slightly reduce the internal friction angle of the specimen. As the nano-clay content increases, the cohesion first decreases and then increases, while the internal friction angle continues to decrease. The improvement effect of fibers and nano-clay depends on their interaction strength with recycled aggregates, including cohesion and friction. When fibers are individually incorporated into recycled aggregates, although the friction angle and friction force both decrease, the increase in cohesion can compensate for this deficiency, so fibers can improve the shear strength of recycled aggregates. When fibers and nano-clay are mixed and incorporated into recycled aggregates, under the action of confining pressure, the friction force between the three is smaller than the occlusal friction force between the recycled aggregates particles. As the nano-clay content increases, the interfacial friction force gradually decreases, and the cohesion increases significantly. Nano-clay improves the shear strength of fiber-reinforced recycled aggregates under the confining pressures of 100 kPa and 200 kPa, which is achieved by increasing its cohesion [45].

## 5. Conclusions

Through unconfined compression tests and unconsolidated undrained shear tests, the compressive strength, failure modes, and shear strength parameters of RA and PNCRA specimens were analyzed, and the following conclusions were drawn:

(1) A fiber content of 0.6% enhances the compressive strength, residual strength, and ductility of recycled aggregates. Furthermore, the performance of the fiber-reinforced recycled aggregate is further improved by adding nano-clay. When the fiber content reaches 6%, both the compressive strength and residual strength are maximized. Additionally, the incorporation of fibers and nano-clay improves the failure morphology of recycled aggregates.
(2) Under the confining pressures of 100 kPa and 200 kPa, the deviatoric stress strength of PNCRA specimens significantly increases after the addition of nano-clay with an optimal amount of 8%. However, under the confining pressures of 300 kPa and 400 kPa, there is no significant improvement in the deviatoric stress strength observed upon the addition of fibers and nano-clay to recycled aggregates.
(3) In terms of shear strength enhancement, the addition of fibers and nano-clay reduces the internal friction angle of the recycled aggregate, but increases its cohesion. In addition, as the nano-clay content increases, so does cohesion.

It should be noted that this experiment only focuses on investigating the improvement of nano-clay and polypropylene fiber on the static load of recycled aggregates without considering dynamic loads such as dynamic triaxial tests, hollow torsional shear tests, resonant column tests, etc. These aspects will also be explored in future research.

## Figures and Tables

**Table 1:** Physical property indexes of the recycled aggregate.

Natural Moisture Content/%	Specific Gravity	Liquid Limit/%	Plastic Limit/%	Maximum Dry Density/g/cm^3^	Optimum Moisture Content/%
5	2.69	18.95	10.29	2.04	10

**Table 2:** Quality indicators of nano-clay.

Composition	Appearance	Montmorillonite Content/%	Density/g/cm^3^	Diameter–Thickness Ratio	Stacking Thickness/nm	Moisture Content/%
Montmorillonite Derivatives	Light-Pink Powder	96–98	0.45	200	≤25	2

**Table 3:** Polypropylene fiber parameters.

Fiber Type	Specific Gravity	Diameter/μm	Length/mm	Tensile Strength/MPa	Melting Point/°C	Elastic Modulus/MPa	Elongation/%	Tensile Limit
Bundle Monofilament	0.91	18–48	9	>3850	160–180	>165	10–28	>150%

**Table 4:** Main performance indicators of the polypropylene fiber.

Specimen Type	Water Content/%	Specimen Density/g/cm^3^	Fiber Content/%	Nano-Clay Content/%
RA	10	2.1	0	0
0PNCRA			0.6	0
4PNCRA			0.6	4
6PNCRA			0.6	6
8PNCRA			0.6	8

Note: RA stands for recycled aggregate; the X in XPNCRA stands for nano-clay content; PNCRA stands for polypropylene fiber–nano-clay–recycled aggregate.

**Table 5:** RA and PNCRA shear strength indicators.

Shear Strength Parameters	RA	0PNCRA	4PNCRA	6PNCRA	8PNCRA
Internal Friction Angle φ/°	41.9	40.4	38.9	36.8	36.6
Cohesive Force c/kPa	133	190	178	231	278

## Data Availability

Data are contained within the article.
